# Unusual lesions that distend the knee joint: pictorial essay*

**DOI:** 10.1590/0100-3984.2015.0154

**Published:** 2016

**Authors:** Luana T. Barros de Lima, Eolo Santana de Albuquerque Filho, Laecio Leitão Batista, Talita Peixoto de Moraes, Bruno Perez Guedes Pereira

**Affiliations:** 1 MD, Radiologist at Hospital Universitário Professor Alberto Antunes (HUPAA) - Universidade Federal de Alagoas (UFAL), Maceió, AL, Brazil.; 2 MD, Radiologist, Head of the Residency Program in Radiology and Diagnostic Imaging at Hospital das Clínicas da Universidade Federal de Pernambuco (UFPE), Recife, PE, Brazil.; 3 PhD, Head of the Residency Program in Angiography and Endovascular Surgery at Hospital das Clínicas da Universidade Federal de Pernambuco (UFPE), Recife, PE, Brazil.; 4 MD, Radiologist at Clínica Derbimagem, Recife, PE, Brazil.; 5 MD, Radiologist at the Instituto de Medicina Integral Professor Fernando Figueira (IMIP), Recife, PE, Brazil.

**Keywords:** Knee/pathology, Neoplasms/diagnosis, Computed tomography, Magnetic resonance imaging

## Abstract

The high number of knee imaging exams at radiology clinics, together with the wide variety of knee disorders, calls for expanding the knowledge about the less common lesions seen in routine diagnostic practice. The purpose of this pictorial essay was to illustrate unusual lesions that distend the knee joint, selected by relevance and evaluated with multiple imaging modalities, including X-ray, computed tomography, and magnetic resonance imaging, as well as to perform a brief review of the literature.

## Abstract (pt)

A elevada demanda de exames para avaliação do joelho nos serviços de diagnóstico, bem como a extensa variedade de lesões geniculares, estimulam a ampliação do conhecimento sobre alterações menos frequentes na rotina de diagnósticos. O objetivo deste ensaio iconográfico é ilustrar didaticamente lesões não usuais do joelho com efeito expansivo, escolhidas em razão da relevância clínica e avaliadas por meio de diversos métodos de imagem como radiografia simples, tomografia computadorizada e ressonância magnética, bem como fazer uma breve revisão da literatura.

## INTRODUCTION

A number of recent studies conducted in Brazil have highlighted the importance of imaging methods in the evaluation of diseases affecting the musculoskeletal system^(1-9)^. The knee is the site of a variety of changes, particularly degenerative and traumatic changes such as fractures and meniscal tears, as well as osteochondral and ligament lesions, which are usual in routine diagnostic practice. However, the high demand for imaging exams to evaluation this articulation calls for knowledge of less common knee lesions, such as pseudotumors and tumors (malignant and benign), such as intracapsular chondroma and lipoma arborescens. All of these diseases have their own radiological approach, which is essential for the diagnostic rationale, which prompts a discussion of their aspects.

## SYNOVIAL CHONDROMATOSIS

First described in 1813, synovial chondromatosis results from chondroid metaplasia of synovial joint tissue and can occur along tendon sheaths as well as synovial bursae^(10)^. It can be primary or secondary to mechanical or inflammatory stress^(11)^. Although it is a benign neoplastic process, it can be aggressive, even evolving to extracapsular involvement^(10,11)^. Malignant degeneration to chondrosarcoma occurs in 5% of cases of primary synovial chondromatosis^(10)^.

**Figure 1:**
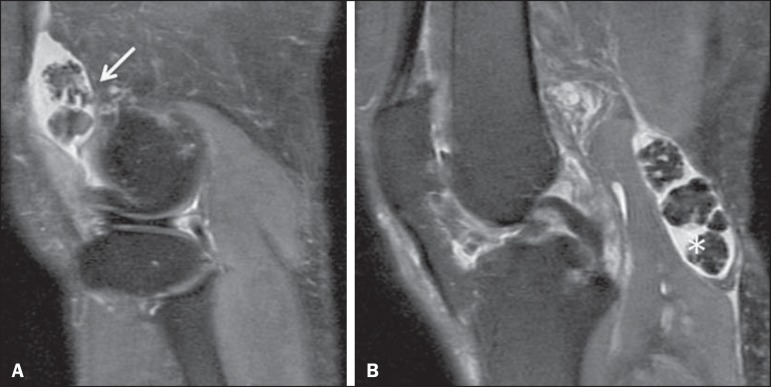
Synovial chondromatosis. Sagittal proton density (PD)-weighted MRI scans with fat suppression. Multiple intra-articular loose bodies located in the suprapatellar recess (arrow) and posterior recess (asterisk), where a popliteal cyst (Baker's cyst) can be seen.

Synovial chondromatosis occurs most commonly in men and in a single joint. The knee is the main site, followed by the hip^(10,11)^. The imaging findings on computed tomography (CT) and magnetic resonance imaging (MRI) are often pathognomonic, including lobular synovitis and multiple intra-articular loose bodies, with a pattern of chondroid calcification, initially adhered to the synovium^(10)^ with subsequent detachment. There can also be erosion of the articular bone surfaces^(11)^. In MRI, loose bodies show hypointense signals on T1-weighted sequences and hyperintense signals on T2-weighted sequences, due to the high amount of water in cartilage tissue, with foci of signal reduction, corresponding to calcifications (Figure 1).

The previously used term osteochondromatosis does not always reflect the histological findings, given that chondral lesions may or may not present endochondral ossification^(10)^. Surgical treatment consists of synovectomy with debridement and excision of the loose bodies^(10,11)^. There have been reports of postoperative recurrence, which could be attributed to the fact that there is synovial tissue remaining^(11)^.

## INTRACAPSULAR CHONDROMA

Bone enchondromas constitute the second most common benign bone neoplasm, following osteochondroma in frequency. Enchondroma is a relatively common incidental finding, seen in 3.3% of knee imaging exams^(12)^. Extraskeletal chondromas, however, are uncommon benign neoplasms that can be divided into three types^(13,14)^: synovial chondromatosis, soft tissue chondroma, and para-articular chondroma. Intracapsular chondromas belong to the third group and are benign tumors arising from chondroid metaplasia of connective tissue of the joint capsule^(14)^. They initially consist solely of hyaline cartilage and endochondral ossification can occur later, which justifies the use of the term osteochondroma as a synonym^(13)^.

**Figure 2:**
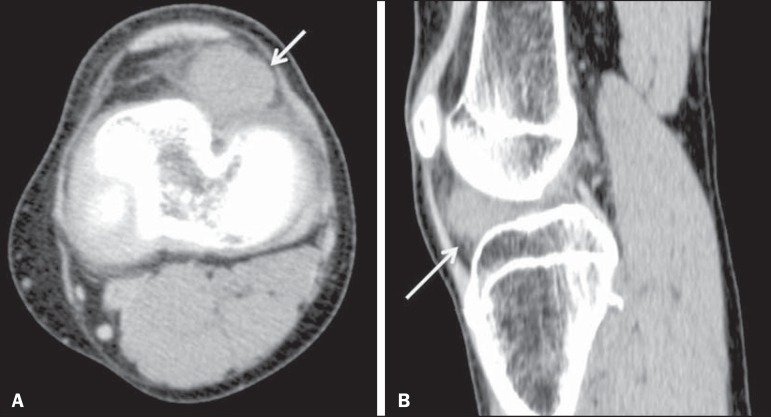
Intracapsular chondroma. CT scans acquired in the axial (**A**) and sagittal (**B**) planes. Expansile lesion with attenuation of soft tissues in the inferior and lateral regions to the patella, together with increased density of the infrapatellar (Hoffa's) fat pad.

**Figure 3:**
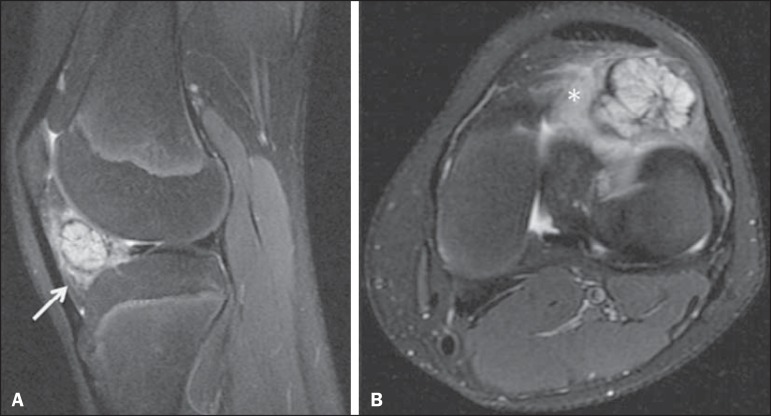
Intracapsular chondroma. PD-weighted, fat-suppressed MRI scans of the same patient depicted in Figure 2, acquired in the axial (**A**) and sagittal (**B**) planes. Heterogeneous expansile lesion (arrow) predominantly hyperintense with loculated borders associated with severe edema of the infrapatellar (Hoffa's) fat pad (asterisk), although with no signs of involvement of the adjacent bone.

The knee is the joint most often affected, typically in the inferior or medial patella, within the extrasynovial compartment^(13,14)^. The appearance on CT is of a mass with attenuation of soft tissues, which may or may not be calcified according to the degree of endochondral ossification (Figure 2). In MRI, areas of cartilage show hypointense signals on T1-weighted sequences and hyperintense signals on T2-weighted sequences, whereas areas of ossification are hypointense on T1-weighted sequences and markedly hypointense on T2-weighted sequences (Figure 3).

## LIPOMA ARBORESCENS

Initially described by Hoffa^(15)^, lipoma arborescens is a benign non-neoplastic condition, consisting of distension of the synovium by villous fat deposits, the macroscopic appearance of which is frond-like^(15,16)^. Although the primary (idiopathic) form has been described, the secondary form is more common and can occur in various types of inflammatory synovitis, such as rheumatoid arthritis. There is a significant association between lipoma arborescens and osteoarthritis, especially in the elderly, because chronic irritation is the mechanism that triggers the synovial response. Although there have been reports of involvement in various joints, the knee is the most common site^(15,16)^.

**Figure 4:**
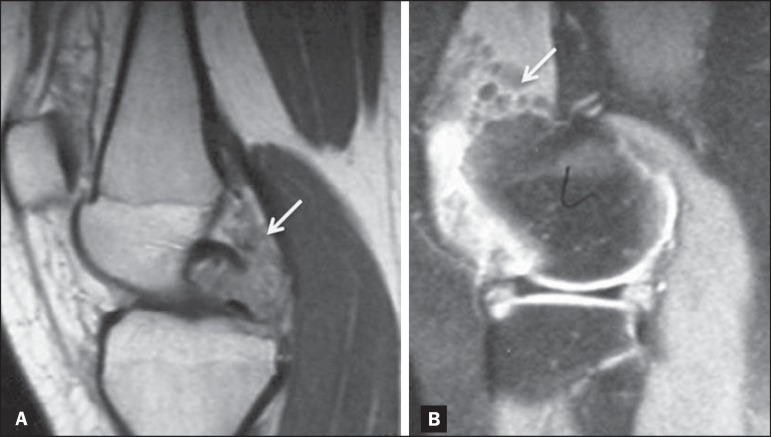
Lipoma arborescens. PD-weighted MRI sequences, both in the sagittal plane, with fat suppression (**A**) and without (**B**), showing intra-articular anomalous tissue, with a frond-like aspect, presenting a hyperintense signal in the PD-weighted sequences without fat suppression and showing a reduction in the signal strength when fat suppression is applied. Moderate joint effusion can also be seen.

**Figure 5:**
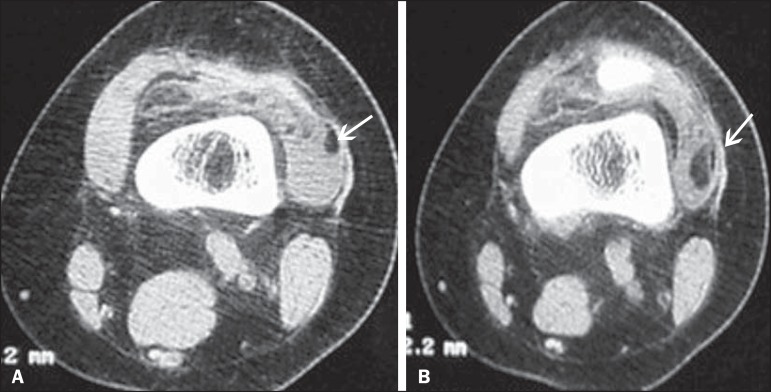
Lipoma arborescens. CT scans, both acquired in the axial plane. The anomalous tissue cited in Figure 4 shows hypoattenuation, indicating its adipose nature (arrows). Joint effusion can also be seen.

MRI can facilitate the diagnosis, demonstrating a frondlike synovial mass that is isointense in relation to fat, an aspect considered pathognomonic (Figures 4 and 5). Lipoma arborescens is often associated with joint swelling and degenerative changes such as meniscal tears^(15)^. Treatment is by arthroscopic synovectomy, and recurrence is uncommon^(16)^.

## PIGMENTED VILLONODULAR SYNOVITIS

Pigmented villonodular synovitis is characterized by benign synovial proliferation, which can arise not only from the joint synovium but also from synovial bursae and tendon sheaths, and can be classified as intra- or extra-articular^(17)^. From a clinical perspective, the symptoms are local pain and swelling, as well as impaired mobility^(17,18)^, affecting a single knee in 80% of cases^(17)^. The term pigmented is applied because of recurrent hematogenous effusion, as evidenced by hemoglobin degradation products.

**Figure 6:**
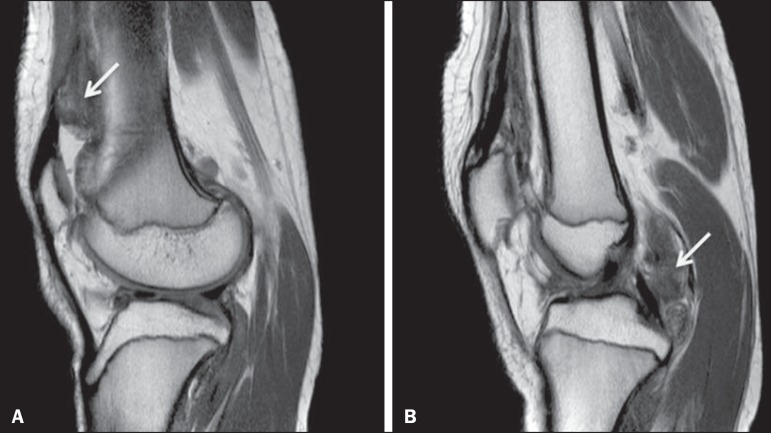
Pigmented villonodular synovitis. PD-weighted MRI sequences, both in the sagittal plane, showing nodular synovial thickening interspersed with hypointense foci, especially in the suprapatellar and posterior recesses of the knee.

Findings in the various methods depend on the subtype of pigmented villonodular synovitis, which has extra-articular, intra-articular, diffuse, and localized forms. It manifests as a mass with soft tissue density, usually with no signs of subjacent bone involvement. In approximately 20% of cases, usually in congruent joints such as the ankle and elbow, bone erosion may occur, which tends to be extrinsic and have sclerotic borders, possibly deriving from the activity of proteolytic enzymes^(17,19)^. Joint swelling and edema of periarticular soft tissue are common. Bone rarefaction, joint degenerative changes, and intra-articular loose bodies are less common, being more common in the diffuse form of the disease^(19)^. MRI provides better tissue differentiation, allowing identification of membranous synovial proliferation and joint effusion (Figure 6). The definitive diagnosis is made histopathologically, and the treatment consists of synovectomy, arthroplasty being reserved for cases in which there is extensive bone erosion^(18)^.

## SYNOVIAL SARCOMA

Synovial sarcoma represents approximately 2.5-10.0% of malignant soft tissue tumors^(20,21)^, preferably affects individuals between 20 and 40 years of age, and most often affects the knee^(21)^. In many cases, it originates from adjacent tendon sheaths and bursae, although it can occur at sites without synovial tissue. Only 5.0-10.0% of cases have an intra-articular component^(21)^, and synovial sarcoma primarily originating from bone is extremely infrequent^(20)^.

The diagnostic imaging of synovial sarcoma represent a challenge due to the absence of a specific pattern. In 50% of cases, an X-ray will show no changes, although calcified foci, generally eccentric and peripheral, are seen on X-rays in approximately 30%. Involvement of subjacent bone is uncommon, and, when it occurs, erosion has an indolent pattern, with signs of aggressive invasion in only 5% of cases. The appearance on CT is of a heterogeneous lobular mass with areas of cystic/necrotic degeneration and post-contrast nodular enhancement^(20,22)^.

**Figure 7:**
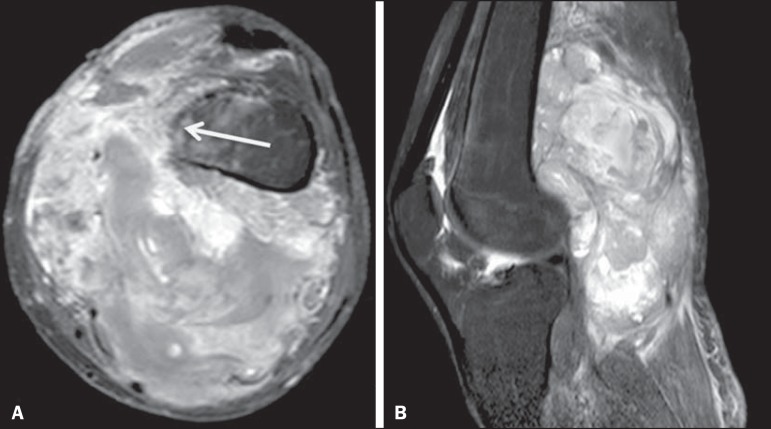
Synovial sarcoma. Fat-suppressed T1-weighted MRI sequences in the axial (**A**) and sagittal (**B**) planes, after intravenous injection of paramagnetic contrast. Voluminous multiloculated expansile lesion, with the "bowl of grapes" aspect, showing intense enhancement after contrast administration. The femoral cortical bone (arrow) is poorly defined.

MRI is the method of choice for evaluating synovial sarcoma, because it provides information on the extent of the tumor and involvement of the neurovascular bundle^(21,22)^. The lesion usually has multiloculated aspect and a heterogeneous signal on T1-weighted sequences, a triple-intensity signal, characterized by interposed areas of hyperintensity, isointensity, and hypointensity, on T2-weighted sequences having been described. Areas of hemorrhage and cystic degeneration are common and can have a "bowl of grapes" aspect, as well as forming liquid-liquid levels (Figure 7).

## ANEURYSMAL BONE CYST OF THE PATELLA

Patellar neoplasms are rare conditions, the most common being chondroblastoma. An aneurysmal bone cyst of the patella is an even less common type of benign neoplasm, which was first described by Jaffe and Lichtenstein in 1942. It commonly occurs in young women and can be primary or secondary to trauma, chondroblastomas, giant cell tumors, or even osteosarcomas^(23)^.

**Figure 8:**
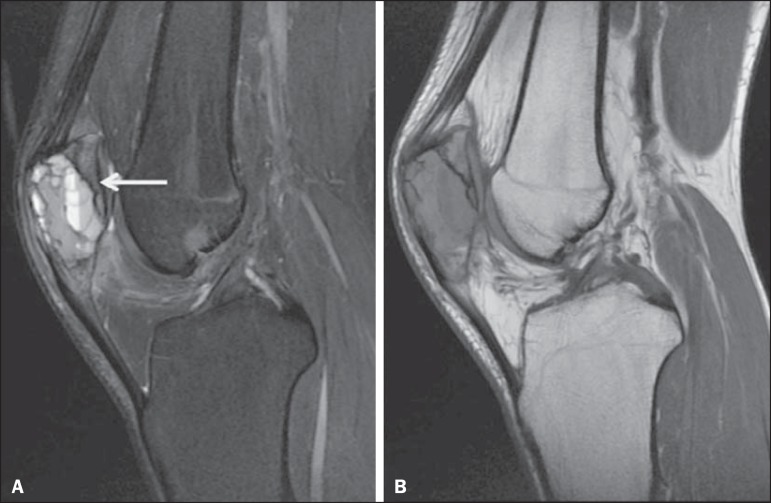
Aneurysmal bone cyst of the patella. PD-weighted MRI sequences, both in the sagittal plane, with fat suppression (**A**) and without (**B**), showing heterogeneous multiloculated expansile lesion involving the patella. In the fat-suppressed sequences, fluid-fluid levels can be seen in some locules (arrow).

On CT, an aneurysmal bone cyst of the patella appears as an expansile, multiloculated lytic lesion. On MRI, the lesion can show hyperintense content on T2-weighted sequences and fluid-fluid levels can typically be seen (Figure 8).

## POPLITEAL ARTERY PSEUDOANEURYSM SECONDARY TO TIBIAL OSTEOCHONDROMA

Osteochondroma is the most common benign bone tumor and accounts for approximately 20-25% of benign bone lesions. It originates from the growth plate and consists of medullary and cortical bone, surrounded by a hyaline cartilage cover^(24,25)^. It predominantly affects men and is solitary in 90% of cases^(25)^. When multiple osteochondromas occur, they are associated with hereditary multiple exostoses, an autosomal dominant disorder^(24)^.

The fixed position of the popliteal artery in the adductor canal, together with the high prevalence of osteochondroma at this site, favors the development of pseudoaneurysm of the popliteal artery, which, albeit a rare condition, is the most common vascular complication of osteochondroma^(24,25)^. It is noteworthy that thrombosis or rupture can complicate a pseudoaneurysm, which makes early diagnosis and treatment important. The differential diagnosis includes the formation of a synovial pseudobursa, as a secondary reaction to osteochondroma, which is distinct from a pseudoaneurysm in that it lacks a relationship with the popliteal artery, shows standard peripheral contrast enhancement as well as no detectable Doppler flow. Factors that can pose challenges in the differential diagnosis include complications of the two diseases, such as bleeding in pseudobursae and thrombosis in pseudoaneurysms.

**Figure 9:**
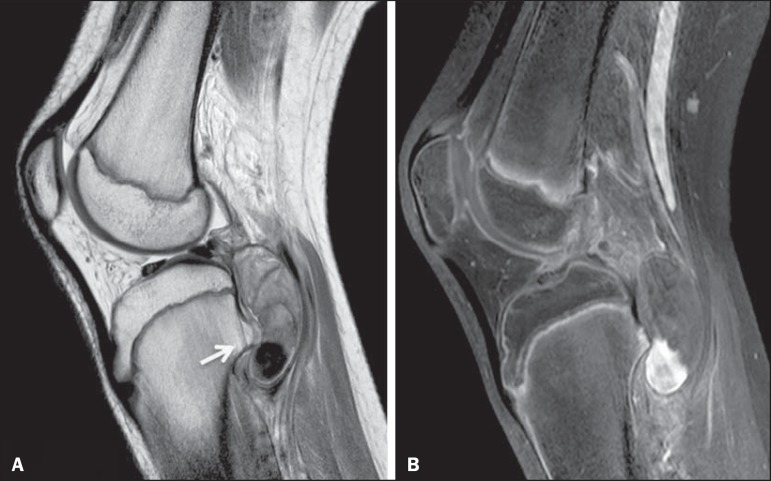
Popliteal artery pseudoaneurysm. Sagittal PD-weighted MRI sequence (**A**) and sagittal fat-suppressed T1-weighted MRI sequence obtained after intravenous injection of paramagnetic contrast (**B**). Small bone projection from the tibial metaphysis (arrow) into the popliteal fossa, where there is an oval image with heterogeneous content, which shows partial enhancement by contrast, representing a partially thrombosed pseudoaneurysm.

Arteriography and Doppler ultrasound are the main methods for the diagnosis of pseudoaneurysm, and conventional X-ray can be used in the diagnosis of osteochondroma. Contrast-enhanced CT and MRI can also aid in the diagnosis and surgical planning (Figure 9).

## CONCLUSION

The variety of lesions that affect the knee is extensive, as is the demand for imaging exams to evaluate this articulation in the diagnostic imaging services, factors which call for expanding the knowledge of unusual disorders in the everyday practice of radiologists.
